# The Correlation between Impacted Third Molar and Blood Group

**DOI:** 10.1155/2021/2775913

**Published:** 2021-11-12

**Authors:** Hanie Ahmadi, Alireza Ebrahimi, Farhad Ghorbani

**Affiliations:** ^1^Student Research Committee, Shiraz University of Medical Sciences, Shiraz, Iran; ^2^Department of Oral and Maxillofacial Surgery, School of Dentistry, Shiraz University of Medical Sciences, Shiraz, Iran

## Abstract

**Background:**

Blood type is among the most important genetical characteristics of any individual and is shown to be correlated with the development of a variety of illnesses including dental diseases. Finding the association of ABO and Rh blood groups and impacted tooth is important in order to predict which population is more vulnerable to grow impacted third molars that could lead to making better intervention. The present investigation tried to take a small step in that regard, by evaluating the correlation between ABO and Rh blood groups and the most commonly impacted tooth, bony impacted third molars among Iranian individuals.

**Methods:**

The investigation was done retrospectively on patients who were referred to the Department of Oral Radiology, Shiraz University of Medical Science, Shiraz, Iran. The patients were classified according to their blood groups, and 40 patients were randomly selected for each blood type. Therefore, the impaction of their third molars was evaluated, and statistical analyses were done in order to find any association.

**Results:**

A total of 320 participants contributed to the study; 136 (42%) were males and 184 (57%) were females. The mean age was 29 ± 6 years. Among all participants, 205 (64%) had no impacted third molar, 26 (8%) had one impacted third molar, 43 (13%) had 2 impacted third molars, 5 (1%) had 3 impacted third molars, and 41 (12%) had 4 impacted molars.

**Conclusion:**

According to the results of the present study, nearly one out of three individuals has at least an impacted third molar in the Iranian population, being more prevalent in individuals between 20 and 30 years old. The evaluation of the relationship between the blood group and impacted third molar revealed that blood groups have no association with the impacted third molars. However, more studies with higher and diversified participants should be done to find comprehensive results.

## 1. Background

Impaction occurs when one tooth or more teeth cannot completely erupt to the normal position in the oral cavity [1–5]. The prevalence of this condition is deemed to be higher in the maxilla and in women [6, 7]. Besides, the chances of becoming impacted are higher for third molars, followed by second mandibular premolar and then second mandibular molar [1, 4]. The expected age for the eruption of third molars is 17 to 21 years; however, this age merely diverges in different races [8]. Furthermore, it has been observed that more than 75% of wisdom tooth still impacted after this time [8].

Congenital problems and genetic diseases, including cleidocranial dysplasia and primary failure of eruption, are the most significant causes of impaction. Besides, as mentioned in previous studies, a significant amount of the impacted third molars might be related to genetic factors [9]. Local and environmental factors, such as malposed and ankylosed teeth, premature loss and retained deciduous teeth, the discrepancy between jaws, tooth crowding, wrong eruption, and tooth bud rotations, are among other causes of tooth impaction. Moreover, some pathologies such as cyst or odontoma could barrier the tooth eruption, leading to impaction [1, 3, 4, 6, 10, 11].

There is no definite gold standard for the management of impacted third molars and defining when to remove third molars preventively. It seems that providing a comprehensive guideline to reduce the unnecessary removal of third molars is required, as these teeth are important to replace with adjacent missing teeth [12]. Hence, conservative follow-up, orthodontic interventions should be considered as well, and extraction of the teeth must be done only in the cases of oral pain, infection, resorption, or cyst [7, 13]. Conversely, many dentists still tend to remove asymptomatic third molars, while a few pieces of evidence support that prophylactic extraction of impacted third molars leads to a decrease in future pathological complications. It should be noted prophylactic surgical intervention to remove impacted third molars should not be done unless it is indicated since the chances of developing pathological conditions in or around follicles of third molars, in contrast to the common belief, seems to be very low. Besides, removing the teeth was not found to be cost-effective, and it has been noted that the economic status and health risk evaluation of patients should be examined before the surgical removal of the tooth [14].

Retention of third molars can deteriorate the periodontal status of the distal surface of the second molar and overall oral health, and consequently is related to increases in the serum interleukin-6, soluble intracellular adhesion molecule-1, and C-reactive protein levels [15]. An untreated impacted tooth may lead to several sequelae, for example, damage to the distal surface of adjacent teeth, crowding of teeth, periodontal and pulp diseases, temporomandibular joint disorders, and oral and maxillofacial cysts and tumors [16–18]. Hence, early diagnosis and treatment of the condition should be done for individuals coming to dental clinics.

Previous studies showed surgical extraction of the third molar lowered lipid fraction and increased systematic inflammation after one week; besides, oxidative stress was reduced after three months, although retention or removal of the third molar did not influence the endothelial function [19]. Moreover, some inflammatory markers, including, leukotrienes, thromboxane A2, prostacyclins, and prostaglandins were released after extraction, leading to an increase in vascularization, vascular permeability, and inflammation. This inflammatory process sometimes could result in several complications such as edema, pain, and trismus [20]. The management of these symptoms includes the administration of corticosteroids and nonsteroidal anti-inflammatory drugs (NSAIDs) through their analgesic and anti-inflammatory effects [21]. Recent findings also mentioned that phytotherapeutic drugs containing an herbal extract mixture of bromelain, baicalin, and escin could be prescribed for relief of the swelling and pain after the removal of the third molar [21].

Blood type is an inherited factor, which is shown to be associated with a variety of diseases [22]. ABO classification is the most common type of categorizing blood types. This classification is consisted of four subtitles termed according to the presence or absence of A and B antigens on the hemoglobin [23] as individuals with blood type O neither having A nor B antigen on their red cells, with blood type AB having both A and B antigens, with blood type A having an A antigen, and with blood group B having B antigen [22]. Another routinely used blood group classification is the Rhesus (Rh) system, categorizing the groups according to the presence of *R* protein in hemoglobin [24]. If a red blood cell presents Rh antigen, it will be termed Rh-positive and if does not, it will be named Rh-negative, respectively [25].

Considering the dental diseases, previous investigations mentioned that there might be associations between the blood types and periodontal diseases and dental caries [23]. In this regard, the evidence showed that the individuals with blood group A are at lower risk of developing dental caries; besides, children with the AB blood group are more vulnerable to develop early childhood caries [23, 26].

By all means, these pieces of evidence that show ABO blood groups could play a diagnostic role and a prognostic factor for oral and dental diseases are still controversial and inconsistent. These controversial results could have been caused by different study methodologies, as well as dissimilar geographical and genetic influences of blood groups in diverse populations [27]. It is important to implement comprehensive studies to statistically determine whether the blood group types and oral diseases are associated; however, few steps have been taken to achieve the mentioned goal till today. Finding the association of ABO and Rh blood groups and impacted tooth is important to predict which population is more vulnerable to grow impacted third molars, which could lead to making better intervention. The present investigation tried to take a small step in that regard, by evaluating the correlation between ABO and Rh blood groups and the most commonly impacted tooth, bony-impacted third molars among Iranian individuals.

## 2. Methods

### 2.1. Population

This cross-sectional study was done on all patients who were referred to the Department of Oral Radiology from March 2019 to May 2020. All patients who came to obtain panoramic and cone-beam computed tomography (CBCT) imaging for examination or dental procedure were considered as the study population. Then, 40 subjects for each blood group type and a total of 320 subjects were randomly selected to be enrolled in this study. These numbers were suggested by an expert statistician to reduce the bias related to the frequency of blood group types in different populations.

Inclusion criteria were individuals with an age between 20 and 80 years old, having high-quality panoramic radiograph or CBCT images and who wanted to participate in the study voluntarily. The exclusion criteria were a diagnosis of any systemic diseases such as cancer, autoimmune diseases such as systemic lupus erythematosus, blood diseases such as thalassemia, congenital syndromes such as Down syndrome, craniofacial deformities such as facial cleft, jaw pathology lesions, primary teeth dentition, and/or previous craniofacial trauma. Besides, the patients with a history of dental extraction and lack of information about their blood groups were excluded from the study, as well.

### 2.2. Study Design

A single operator and radiographic device were assigned to take the radiographs. The participants were interviewed and examined by a single dental practitioner (author). Clinical oral examinations, X-ray panoramic, and CBCT images investigation were undertaken to diagnose bony-impacted teeth. A separate survey containing information on sex, age, blood group type, and the number of impacted teeth (if there are) was used as a record for each participant. If the participants had impacted teeth, the number and the site of teeth were recorded on the survey sheet. Blood groups of patients were obtained from their medical records, their blood and organ donation cards, and driving license cards.

### 2.3. Ethical Considerations

The study was completely explained to each participant, and signed informed consent was obtained. The study protocol was approved by the Medical Ethics Committee of Shiraz University of Medical Sciences by the Institutional Review Board (IRB) (number IR.SUMS.DENTAl.REC.1399.080).

### 2.4. Statistical Analysis

Descriptive statistics including mean, standard deviation, and frequency were measured. A Chi-Square test was applied to determine the correlation between ABO blood groups and the prevalence of impacted teeth. All the statistical analyses were performed using Statistical Package for the Social Sciences (SPSS version, 23), and *P* values < 0.05 were considered significant.

## 3. Results

Among a total of 320 participants who were involved in the study, 136 (42%) of them were males and 184 (57%) were females. The mean age of participants was 29 ± 6 years, classified into six age groups as under 20, 20 to 29, 30 to 39, 40 to 49, 50 to 59, and greater than 60 years with the number of patients equal to 2 (0.6%), 169 (52.8%), 126 (39.3%), 18 (5.6%), 3 (0.9%), and 2 (0.6%), respectively (Table 1).

**Figure 1:**
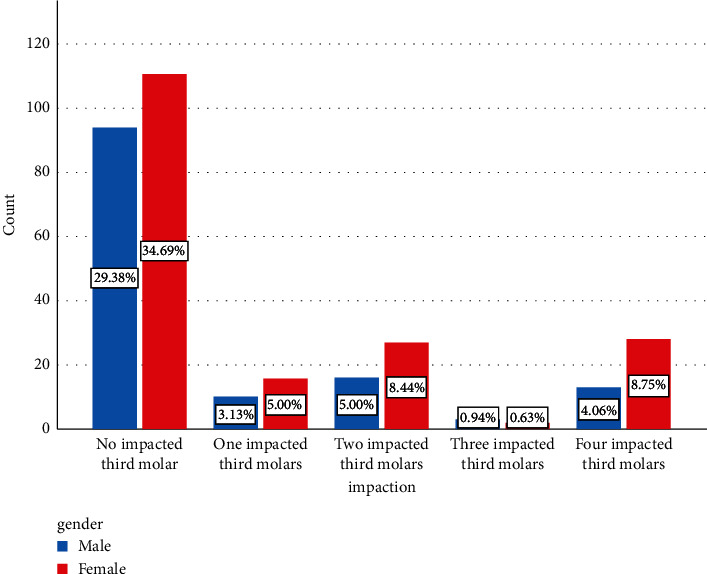
Frequency distribution of the impacted third molar and genders.

Considering the impacted third molar, 205 participants (64%) did not have any impacted ones, 26 (8%) had one, 43 (13%) had two, 5 (1%) had three, and 41 (12%) had four impacted third molars. Women had a higher prevalence of the impacted third molar, as 73 (39%) of the female participants and 42 (30%) of the male participants had at least one impacted third molar.  Figure 1 shows that more females were diagnosed with having impacted third molars; however, there was no significant association between gender and incidence of the impacted third molar (*P* > 0.005).

Our data showed that the subjects with no impacted third molar in the blood groups A, B, AB, and O were distributed as 51 (24%), 51 (24%), 51 (24%), and 52 (25%). In addition, among subjects with different ABO blood groups, 11 (26%) participants with blood group A, 10 (24%) participants with blood group B, 8 (19%) participants with blood group AB, and 12 (29%) participants with blood group O had all third molars impacted (Table 2). Furthermore, among participants with negative RH, 112 (54%) of them had no impacted third molar and this figure was 93 (45%) for individuals with positive RH (Table 3). Nevertheless, the Chi-Square test showed the association of blood groups and RH factor with the impacted third molar was statistically nonsignificant (*P* > 0.05), which means there was no association between the blood group and RH factor with a prevalence of the impacted third molar.

## 4. Discussion

Third molars are considered to be more prone to be impacted in the oral cavity [10]. The prevalence of the impacted third molar was calculated to be between 16% and 68% in prior investigations [28]. Our study estimated that this number is around 35%, and thus, one out of three people has at least one impacted third molar tooth in the Iranian population, approximately. This estimation is higher than that shown in a study in the Swedish population (30.3%) and is lower than that of studies by Hashemipour et al. and Quek et al. [28–30]. These conflicting results could have happened due to different sampling methods and methodologies as well as dissimilar genetic backgrounds of individuals. In the present study, we showed that there is no significant association between the development of impacted third molars and different blood groups.

Previous studies mentioned that people are more vulnerable to grow the third impaction during their 30s or 40s [28]. However, our results demonstrated that more than half of the patients with impacted third molars were in the second decade of their lives. It has been also noticed that the impaction of the third molar was more common in women [28]. While the present study had found relatively consistent results, we concluded that this seemingly higher prevalence of impacted third molar in women is not statistically significant. Having said that, we should bear in mind the fact that the growth patterns of males and females are fundamentally different, in the sense that women usually stop growing jaw space when the third molars start to erupt, whereas the growth of jaws continues during the time of eruption of third molars in men, creating more space for third molar eruption [31].

Dental practitioners can diagnose impacted teeth while doing routine inspections and examining periapical, occlusal, or panoramic radiographic images [2, 32]. The most ordered type of imaging used to diagnose the impacted teeth is orthopantomography (OPG), since it is commonly fast and low-priced, has lower radiations compared to other means, and illustrates the jaws bilaterally [10]. But, because of the restrictions of two-dimensional radiographs, such as the inability to measure the buccolingual dimensions, some dentists may prefer to use more advanced methods, including CBCT imaging or low-dose computed tomography (CT) [32, 33].

Regardless, the lack of jaw space, as mentioned, is the most common underlying cause of impacted molar [33]. And, as shown in prior investigations, the growth of the jaw and arch dimension is generally controlled by the genes [34, 35]. It may seem feasible to propose that the presence of different types of blood groups, which are deeply related to the genes, could be moderately associated with the incidence of third molar impaction. If so, we then might be able to use blood group type as an indicator for incidence of third molar impaction.

Another aspect to be noticed is that inflammatory mediators increase in the case of pericoronitis associated with third molars, contributing to inflammation, pain, and swelling [36]. While no association was found between the release of cytokines and history of pericoronitis or periodontal diseases [37], it has been reported that the levels of tumor necrosis factor (TNF) *α* and interleukin-1*β* in the gingiva around the pericoronitis are tended to be higher than other areas. Also, interleukin-6 is inclined to be more presented in the bony-impacted third molars rather than tissue-impacted third molars [38, 39].

Former studies demonstrated different blood groups could be considered as risk factors for certain diseases, for example, blood groups A and B were associated with atherosclerosis and blood group A increased the chance of developing cholelithiasis, gastric ulcer, and gastric carcinoma [40–42]. Another study revealed that the individuals with blood group O had a higher risk of developing hypertension [43]. Moreover, individuals with blood groups O and A were more likely to be infected with *Helicobacter pylori* [44]. A relationship between blood types and congenital cataracts in the Asian race was also observed in prior investigations [45, 46]. Furthermore, recent reports mentioned that individuals with blood group A have a greater risk and those with blood group O are at a lower risk of contracting the novel coronavirus disease (COVID-19) [47].

Considering dental and oral diseases, a growing body of evidence suggests that there may be a correlation between the types of blood groups and the incidence of oral diseases such as periodontitis. The first research that showed an association between blood groups and periodontal diseases was done in 1930 [48, 49]. Thereafter, it has been shown that individuals with blood group A are more vulnerable to develop gingivitis and individuals with blood group O are at a higher risk of growing periodontitis [27]. Having blood group B was found as a risk factor for aggressive periodontitis, and having blood group A was found as that for severe alveolar bone loss [50]. Moreover, it has been noticed that advanced forms of generalized periodontitis were more prevalent in people with blood types O and A, compared with blood groups B and AB [51]. Besides, Nikawa et al. reported that denture wearer individuals with blood group O were more prone to denture stomatitis in comparison with those who had other blood groups [52]. It is also noteworthy that previous studies reported that people with blood groups A and B have upper rates of malocclusion and those with blood group B have a greater prevalence of maxillofacial deformities [53, 54].

Conversely to the abovementioned proofs, some studies did not found any association between dental diseases and different types of blood groups. We believe that these sorts of findings are also important in the way to reach systematic and comprehensive conclusions. One example was the investigation that found there is no association between the ABO blood group antigens and localized juvenile periodontitis [55]. Another is the study that evaluated the correlation between the blood group and malocclusion and found no association [56, 57]. Our study is another one that showed although there was a higher percentage of blood group O among patients with impacted third molars, there is no statistically significant association between the types of blood group and impacted third molar.

The results of our study might have been affected by some limitations. One of the most important weaknesses of our study is that all of our participants were Iranian, while different ethnicities should be investigated to estimate the relationship between blood group and impacted teeth. A rising number of participants of both genders, different ages, and blood types are needed to be investigated in this regard.

## 5. Conclusion

According to the result of the present study, nearly one out of three individuals has at least an impacted third molar in the Iranian population, being more prevalent in individuals between 20 and 30 years old. The evaluation of the relationship between the blood group and impacted third molar revealed that blood groups have no association with the impacted third molars. However, more studies with higher and diversified participants should be done in order to find comprehensive results.

## Figures and Tables

**Table 1:** Demographic data.

Age distribution	Number	Percentage
Under 20 years	2	0.6
20 to 29 years	169	52.8
30 to 39 years	126	39.3
40 to 49 years	18	5.6
50 to 59 years	3	0.9
60 to 69 years	2	0.6
Gender		
Male	136	42
Female	184	57

**Table 2:** Frequency of the impacted third molar in blood types.

	Blood group								*P* value
	A		B		AB		O		
	Count	Percentage	Count	Percentage	Count	Percentage	Count	Percentage	
No impacted molar	51	24.0	51	24.0	51	24.0	52	25.0	>0.05
One impacted molar	7	28.0	7	28.0	8	32.0	3	12.0	
Two impacted molars	9	20.0	11	25.0	11	25.0	12	27.0	
Three impacted molars	1	20.0	1	20.0	2	40.0	1	20.0	
Four impacted molars	11	26.0	10	24.0	8	19.0	12	29.0	

**Table 3:** Association between RH factor and impacted teeth.

	RH				*P* value
	−		+		
	Count	Percentage	Count	Percentage	
No impacted third molar	112	54.0	93	45.0	>0.05
One impacted third molar	10	38.0	16	61.0	
Two impacted third molars	19	44.0	24	55.0	
Three impacted third molars	2	40.0	3	60.0	
Four impacted third molars	17	41.0	24	58.0	

## Data Availability

Data are available on request to the corresponding author.
